# Supplementary material for: Implementing psychosocial interventions for teachers’ mental health: Protocol for integrating scoping review with teachers lived experiences in LMICs
Source: PLoS One. 2025 Jan 27;20(1):e0317351. doi: 10.1371/journal.pone.0317351 (PMC11771928; doi:10.1371/journal.pone.0317351)
Supplement: S1 Appendix — (DOCX) [file pone.0317351.s001.docx]

# **S1 Appendix. Search strategy**

The following search strategy has been developed for Scopus:

( ( TITLE ( school W/1 staff ) OR TITLE ( school W/1 personnel ) OR TITLE ( teacher* ) OR TITLE ( educator* ) ) AND ( TITLE-ABS ( psycho* W/1 interven* ) OR TITLE-ABS ( cognitive W/1 behav* ) OR TITLE-ABS ( psychotherap* ) OR TITLE-ABS ( counsel?ing ) OR TITLE-ABS ( mindful* PRE/1 ( interven* OR therapy OR program* ) ) OR TITLE-ABS ( group PRE/1 ( interven* OR therapy OR program* ) ) OR TITLE-ABS ( coping W/1 ( promot* OR interven* OR therapy OR training OR program* ) ) OR TITLE-ABS ( well?being W/1 ( promot* OR interven* OR therapy OR training OR program* ) ) OR TITLE-ABS ( resilience W/1 ( promot* OR interven* OR therapy OR training OR program* ) ) OR TITLE-ABS ( "mental health" W/1 ( promot* OR interven* OR therapy OR training OR program* ) ) OR TITLE-ABS ( "mental health literacy" ) OR TITLE-ABS ( workplace W/1 ( program* OR interven* ) ) OR TITLE-ABS ( ( interven* OR therapy OR management ) W/1 ( "mental disorder" OR "mental problem" OR depress* OR anxi* OR burnout OR stress* ) ) ) ) AND PUBYEAR > 1999 AND PUBYEAR < 2025 AND ( LIMIT-TO ( LANGUAGE , "English" ) )

A total of 1408 records were retrieved from the search conducted on 31 March 2024.
