# Supplementary material for: Implementing psychosocial interventions for teachers’ mental health: Protocol for integrating scoping review with teachers lived experiences in LMICs
Source: PLoS One. 2025 Jan 27;20(1):e0317351. doi: 10.1371/journal.pone.0317351 (PMC11771928; doi:10.1371/journal.pone.0317351)
Supplement: S2 Appendix — (DOCX) [file pone.0317351.s002.docx]

# **S2 Appendix. Data extraction matrix**

The data extraction sheet will include the following variables:

| Citation details |
| --- |
| Document type |
| Study context and methods (country, design used, sample size, comparator type [if any]) |
| Participants details (e.g., average age, gender distribution, ethnicity, average teaching experience and teaching level distribution) |
| Intervention details (e.g., focus [promotion/ prevention/ treatment], underlying theoretical model, mode and format of delivery, duration of intervention, session dosage, type of provider, training of provider) |
| Type of effectiveness outcome assessed for mental health (e.g., well-being, psychosocial distress, and/or determinants of mental health such as mindfulness, emotional regulation, critical thinking, self-efficacy), and/ or mental problems (such as depression, anxiety, burnout, and post-traumatic stress disorder), quantitative and/or qualitative outcome measure used, key findings on measure, relevant participant verbatims |
| Type of implementation outcome assessed (i.e. acceptability, feasibility, fidelity, cost, adoption, appropriateness, penetration) quantitative and/or qualitative outcome measure used, key findings on measures, relevant participant verbatims |
