# Supplementary material for: Implementing psychosocial interventions for teachers’ mental health: Protocol for integrating scoping review with teachers lived experiences in LMICs
Source: PLoS One. 2025 Jan 27;20(1):e0317351. doi: 10.1371/journal.pone.0317351 (PMC11771928; doi:10.1371/journal.pone.0317351)
Supplement: S3 Appendix — (DOCX) [file pone.0317351.s003.docx]

# **S3 Appendix. Topic guide for group discussion with PWLEs**

Each effective and/or successfully implemented intervention package identified from the review will be discussed with the advisory group of teaching professionals with lived experience (PWLE). Interventions with similar characteristics will be grouped to expedite the review process and avoid redundancies. The following questions reflect broad domains of inquiry. Specific verbatim and follow-up questions will be developed after collating the findings of the scoping review.

- **Based on the review findings, what aspects of this intervention did you find favourable or unfavourable?**
- **If you were part of the mental health planning committee at your school, would you recommend this intervention for teachers? Why or why not?**
- **If you could modify one or two aspects of this intervention to make it more suitable for teachers, what would you change and why?**
- **How can we ensure that this intervention is easy to use and accessible for teachers in various settings, such as urban and rural areas, or public and private schools?**
- **Who do you think would be the most appropriate person to deliver this intervention and what support/ training they would need to deliver it effectively?**
- **What challenges are anticipated in implementing this intervention in your school?**
