# Supplementary material for: Implementing psychosocial interventions for teachers’ mental health: Protocol for integrating scoping review with teachers lived experiences in LMICs
Source: PLoS One. 2025 Jan 27;20(1):e0317351. doi: 10.1371/journal.pone.0317351 (PMC11771928; doi:10.1371/journal.pone.0317351)
Supplement: Attachment — Submitted filename: rebuttal plosv1.docx [file pone.0317351.s005.docx]

**Response to Reviewers**

We thank the reviewers for their helpful comments. Our responses are included below. The page and line numbers correspond to the tracked version of the manuscript.

Reviewer #1:

1) General Comments
   The manuscript addresses an important and timely topic: the mental health of teachers and the implementation of psychosocial interventions in low- and middle-income countries (LMICs). The integration of a scoping review with lived experiences is a novel approach that could provide valuable insights. However, the current manuscript has some areas that need clarification. These comments are aimed at improving the clarity, rigour, and overall quality of the manuscript.

   Below are detailed comments and suggestions for each section of the manuscript.
2) Abstract
   a) The abstract should clearly define the integration of lived experiences. While the current description details the scoping review, it is vague about the timing and conduct of the focus groups and how the data will be integrated. This creates the impression that the discussions with teachers will occur in the future and are not part of the study.

**Response**: We have revised the abstract to indicate that the integration of lived experiences will take place following completion of the scoping review. The discussions with PWLEs will consist of four online meetings conducted in a workshop-group discussion format. The process of how these discussions will be conducted is also summarised in the abstract. The revised text is as follows (lines 25-26, 37-43)
**The study combines scoping review with insights from an advisory group of teaching professionals with relevant lived experiences (PWLEs) …**

**A purposively selected sample of 10 PWLEs will form the study advisory group and participate in four online workshop-group discussions. The meetings will include a presentation of the scoping review findings, followed by discussions on the relevance of these intervention packages for LMICs, adaptations needed to make them acceptable among school teachers and feasible for delivery in low-resourced settings like India.** The synthesis of the data will employ narrative and thematic approaches to generate actionable insights for implementing psychosocial interventions in LMICs.

b) The authors refer to the study as a "review" ignoring the fact that this is a mixed-method study that integrates two different methodologies.

**Response**: We have revised the abstract to indicate this study employs dual methodology, integrating a scoping review with discussions involving lived experiences. The revised text is as follows (lines 20-23)

This **mixed methods** study aims to conduct a scoping review of characteristics, effectiveness, and implementation outcomes of psychosocial interventions for teachers' mental health and mental problems, integrating these with teachers' lived experiences to inform the implementation of mental health interventions in LMICs

Similar changes have also been made in the main text. The revised text is as follows (lines 99-105):

The current **mixed methods** study aims to bridge this gap by c**onducting a scoping review** of broad range of evidence on characteristics, effectiveness and implementation outcomes of psychosocial interventions for teachers’ mental health and mental problems. Additionally, by **exploring and incorporating insights from an advisory group** of teachers with relevant lived experiences, it aims to identify implications for providing psychosocial interventions for teachers’ mental health in LMICs, thereby contributing to a more nuanced understanding of how to support this vital workforce effectively..

c) The authors use the terms "mental health and mental health problems." I suggest using "mental wellbeing and mental health symptoms," as most psychosocial interventions for teachers aim to enhance these aspects. These interventions typically address self-reported symptoms of common mental health issues rather than focusing on diagnosed mental health disorders. Please review these terms throughout the manuscript.
**Response:** We have retained the terms "mental health" and "mental problems" as they better align with the conditions covered in this review. These terms are described in detail in the methods section of the main article. We have also updated the definitions in the Methods sections to make the distinction clearer for the reader (lines 152-161), as follows:

Psychosocial interventions for mental health are defined as individual, group, or organizational level programs that are designed to improve mental well-being, **prevent symptoms of psychological distress,** and/or modify its determinants (e.g., emotional regulation, critical thinking, problem-solving). Interventions addressing mental problems are defined as those **preventing worsening of subthreshold or diagnosable** common mental problems such as depression, anxiety, and post-traumatic stress **disorders.**

3) Introduction
   a) The introduction provides a good background on the importance of teachers' mental health.
   **Response:** We appreciate the reviewer’s positive feedback on the clarity of the introduction.
4) Review Questions
   a) The review questions need to be more specific. Clarify what is meant by "characteristics" and "effectiveness" of interventions, as you did in the third question.

**Response:** To address this, we have included definitions of the terms "characteristics" and "effectiveness" after introducing the study objectives. Additionally, we have also included the definitions of “implementation outcomes” and “advisory group.” The revised text is as follows (lines 106-116):

**For the current study, "characteristics" refers to the features of the psychosocial interventions, including the theoretical framework, settings and mode of delivery, duration, session structure, and provider details. "Effectiveness" refers to the impact of these interventions on reducing symptoms of mental problems and/or improving mental health among teachers.** “Implementation outcomes”, as defined by Proctor et al [22], will cover acceptability, feasibility, fidelity, adoption, appropriateness, cost, penetration, and sustainability. **The “advisory group” refers to purposively selected teaching professionals with relevant lived experiences (PWLEs) who will work with the study team and provide critical insights and feedback on the findings from the scoping review.**

b) The second question includes the impact of these interventions on job performance and other functioning-related outcomes. However, one of the study selection criteria states that job-related outcomes (e.g., quality of teaching, teaching efficacy/confidence, or performance in a specific course) will be excluded. I suggest removing the second portion of the second question.

**Response:** To clarify, we intended to exclude articles where the primary focus is on job-related outcomes. However, for the articles where the primary focus is on mental health and mental problems, we were interested in tracking their impact on job outcomes as well. To avoid confusion for the reader and streamline our outputs, we have revised the second review question to remove the impact on job performance and other functioning-related outcomes.

5) Methods
   a) The authors should Include in the context component of the eligibility criteria that only interventions conducted in LMICs will be included.

**Response:** The scoping review aims to cover studies conducted globally to ensure a comprehensive understanding of the subject. Given the limited number of studies from LMICs in previous reviews, we aim to balance this by giving greater weight in our description to intervention programs carried out in LMICs and prioritizing them in discussions with PWLE. Below is the revised version of the manuscript indicating these changes (lines 166-169):

This review will include studies conducted in diverse contexts and settings **worldwide** to ensure a comprehensive understanding of the subject. **In line with the study's objective to develop implications for psychosocial interventions in LMICs, we will give greater emphasis in our final report and in discussions with PWLEs to intervention programs conducted in LMICs.**

b) I suggest narrowing the search period to the past 5 to 10 years to ensure relevance.

**Response:** We have initiated the article screening process, making it challenging to alter the study period at this stage.

c) The authors mention that "data to be extracted includes details about the participants" (line 187). I suggest replacing "details" with "sociodemographic information and job-related variables".

**Response**: In response to your comment, we have made the following changes to the manuscript (line 226):

The data to be extracted include **sociodemographic and teaching related information** (such as average age, gender distribution, ethnicity, average teaching experience, and teaching level distribution);

d) The data analysis and presentation section is a crucial part of this protocol. The current description does not sufficiently emphasize the mixed methods proposed in this manuscript. The authors should first complete the description of the scoping review portion of the study. Then, in a separate section, they should detail the integration of teachers' suggestions. This new section should thoroughly describe the process of creating lay summaries, the number of focus group sessions, their duration, and the specific roles of teachers in these discussions. For instance, will there be a script for conducting the focus group sessions? What questions will the researchers ask? Additionally, clarify whether teachers participating in the focus group sessions will be compensated for their time.

**Response:** We have revised the first paragraph of the methods section to describe the mixed methods approach we are proposing. The revised manuscript text is as follows (lines 135-140):

**The study will used a mixed methods approach that combines a scoping review and group discussions with advisory teaching PWLEs.** To achieve the first three objectives, the Preferred Reporting Items for Systematic Reviews and Meta-Analyses Extension for Scoping Reviews guidelines (PRISMA-ScR, [26]) will be used**. For the fourth objective, a combination of group discussion and workshop format will be employed. The final protocol was registered prospectively on the Open Science Framework.**

Additionally, we have created a new subsection that covers the detailed methodology and data analysis related to discussions with PWLEs. The new section describes in detail the process of gathering teachers' feedback, including the number of group meeting, their duration, format, topic guide for group discussions and approach to data analysis synthesis. The updated text is as follows (lines 243-304):

**Discussions with advisory group of teaching PWLEs**

**Design**

**The lay summary of the scoping review will be discussed with an advisory group of teaching PWLEs using a combination of workshop and group discussion formats. These meetings, four in total, will be conducted online using a secure videoconferencing platform such as Zoom. The duration of each meeting will be determined based on the breadth of content to be reviewed and group members' availability.**

**Participants and recruitment**

**The advisory group will consists of upto 10 school teachers, aged 18 and above, who have been teaching for 6 months or more in senior secondary schools in the National Capital Regions of India. The participants must be proficient in written and spoken English, as needed to participate fully in study procedures. The size of the advisory group is in line with previous research using similar integrative reviews [31-33].**

These PWLEs will be recruited via social media platforms (e.g., Twitter, Instagram, LinkedIn) and through an outreach team connected to our research group or host institution. Interested individuals will be directed to a secure link providing detailed study information and an expression of interest form. This form will inquire about their sociodemographic information, teaching experience, mental health status, access to internet-enabled devices, and availability for consultation meetings. Upon receiving expressions of interest, study team will purposefully select PWLEs to ensure a diverse representation in **terms of age, gender, teaching experience, teaching settings (public/ private schools) and lived experiences of mental health symptoms.**

**Procedures**

**Purposively** selected participants will receive a digital consent form confirming their participation. At this stage, participants will be given the choice to be acknowledged by name and professional affiliation in the acknowledgments section of any future publications, respecting their preference for recognition and highlighting the value of their contributions. Participants who provide consent will be asked to join **online group meetings on selected dates. These meetings will involve presentations on review findings, framed in a language suitable for laypersons,** followed by group discussions to gauge advisors’ perceptions on the following **(i) relevance of the effective and successfully implemented intervention packages, especially those evaluated in LMICs, for the Indian context ; (ii) suggested adaptations needed to make these potentially relevant intervention packages more acceptable among teachers ; and (iii) the practical implications for delivering these interventions in schools or other suitable locations by peer teachers or other potential providers in LMICs. A topic guide is provided in S4 Appendix. The discussion will be audio-recorded and participants will be provided with gift vouchers upon completion of the study. The amount of each voucher will be decided as per the guidelines of the host institute of the first author.**

**Data analysis**

**The recordings will be transcribed, and transcripts will be pseudonymized to respect the PWLEs' confidentiality and right to privacy. Once transcribed, transcripts will be cleaned and analysed thematically.** The final stage of synthesis will involve consolidating findings from both the scoping review and discussions with PWLEs**. We will identify areas of convergence, where both the literature and teaching PWLEs agree, and areas of divergence, where they differ with respect to the potential for implementation and impact.** This comprehensive synthesis will inform recommendations for providing psychosocial interventions aimed at improving teachers' mental health in LMICs, **specifically** in India.

e) How did the authors determine the number "8 - 10 teachers"? How will the authors ensure equitable and diverse representation of teachers with different backgrounds in this group (e.g. sex, gender, ethnicity, geographic origin, teaching grade/subject/school)?

**Response:**  We have revised the manuscript (lines 255-256) to indicate that **the ‘size of advisory group is in line with previous research using similar integrative reviews [31-33].’** Further, we will purposively sample from the pool of applicants to ensure diverse representation in terms of age, gender, teaching experience, teaching settings (public/private schools), and lived experiences of mental health symptoms (lines 263-265).

We have acknowledged the small size of advisory group as a limitation in the discussion section (lines 347-352)

Additionally, recruitment via social media and **the small number of English-speaking advisory PWLEs** might cause selection bias, possibly not fully representing the diverse teacher experiences in LMICs. **Despite these challenges limiting the generalizability of the findings, the strengths of this study lie in its integration of results from multiple sources to develop a comprehensive set of guidelines for** future research on **adapting** and implementing these interventions for LMICs.

f) Replace "self-identified mental health concerns" with "lived experiences of mental health symptoms"(line 204). Keep consistency in these terms throughout the manuscript, taking into account that psychosocial interventions do not always target diagnosed mental health disorders, but mental health symptoms.

**Response:** We have replaced "self-identified mental health concerns" with "lived experiences of mental health problems" (line 265).

g) In lines 220 to 223, the authors outline their goals for the discussions with teachers. However, these themes—such as the suitability of interventions, policy changes, and future research—are ambitious and complex for a lay audience. I suggest revisiting this paragraph and adopting a more straightforward script for conducting the focus group sessions.

**Response:** We have simplified the goals for the discussions with teachers to ensure they are clear and understandable. The revised goals are to gather teachers' perspectives on following (lines 287-292):

**(i) relevance of the effective and successfully implemented intervention packages, especially those evaluated in LMICs, for the Indian context ; (ii) suggested adaptations needed to make these potentially relevant intervention packages more acceptable among teachers ; and (iii)** the practical implications for delivering these interventions in schools or other suitable locations by **peer teachers or other potential providers in LMICs.**

h) There is a typo in line 226 "scopingreview".

**Response:** Corrections made (line 300)

6) Discussion
a) Clearly articulate the practical implications of the scoping review findings for implementing psychosocial interventions in LMICs and emphasize the contribution of integrating teachers' lived experiences to the relevance and applicability of the findings, as the novelty of this protocol.

**Response:** We have highlighted the use of mixed methods as one of the novel features of this study. We have also clearly articulated the practical implications of the scoping review and how integrating insights from teachers' lived experiences will enhance the applicability of findings in LMICs. The revised text is as follows (lines 306-342):

**The novelty of this protocol lies in its mixed methods integrative approach, combining a systematic review with lived experience consultations. This scoping review, set to complete by mid-2025,** will provide a comprehensive synthesis of evidence on **psychosocial interventions for teachers' mental health and mental problems, across various contexts, intervention types and outcomes.** By integrating teachers' perspectives with scoping review, we will be able to shed light on unique needs of teachers in LMICs like India and develop guidelines for contextually appropriate interventions to improve teachers mental health**, an area often overlooked in previous reviews on this topic [15-21]. Furthermore, insights from the review and advisory group can guide the development of training programs for counsellors and educators, ensuring they are equipped to deliver these interventions effectively. This integrative approach not only enriches the study** findings but also supports patient-centered and participatory research methodologies, recognized for their potential to improve the relevance and impact of research outcomes [34].
