# Supplementary material for: Implementing psychosocial interventions for teachers’ mental health: Protocol for integrating scoping review with teachers lived experiences in LMICs
Source: PLoS One. 2025 Jan 27;20(1):e0317351. doi: 10.1371/journal.pone.0317351 (PMC11771928; doi:10.1371/journal.pone.0317351)
Supplement: Attachment — Submitted filename: Rebuttal letter.docx [file pone.0317351.s006.docx]

Dear Editor and Reviewers,

We sincerely appreciate the time and effort you have invested in reviewing the manuscript. We have carefully considered each point raised and have made the necessary revisions to address your concerns. Our responses and modification to the manuscript are described below, with the page and line numbers referring to tracked version:

### **Reviewer #1:**

*The authors have thoroughly addressed all the reviewer’s comments and suggestions. No further revisions are needed.*

**Response:**

Thank you for your positive feedback. We are pleased that our revisions have met your expectations and that you find the manuscript satisfactory.

### **Reviewer #2:**

*Congratulations to the authors for embarking on this interesting study. I have a few comments for the authors to address which hopefully improve the quality and understanding of the entire study.*

#### **Comment 1:**

*The decision to exclude studies focusing on job-related outcomes (e.g., teaching quality, efficacy) may overlook the interconnectedness between mental health and job performance. Mental health interventions often impact job-related outcomes, and excluding these may lead to an incomplete understanding of the benefits of psychosocial interventions.*

**Response:**

Thank you for your comment. While we acknowledge that mental health and job performance are interconnected, this review focuses specifically on mental health outcomes to provide a detailed analysis of psychosocial interventions in this domain. The impact on job-related outcomes, although important, is beyond the scope of this review but represents a valuable area for future research. We will keep this in mind for future secondary analyses that may arise from this work, where we can delve deeper into the impact of psychosocial interventions designed for mental health on job-related and other functional outcomes.

#### **Comment 2:**

*Given your review topic, it would have been more suitable to use the PICO format (P-population, I-intervention, C-comparison, O-outcome) to develop your search strategy and eligibility criteria.*

**Response:**

We appreciate your suggestion regarding the use of the PICO framework. In designing our scoping review, we considered the pros and cons of both the PICO and PCC (Population-Concept-Context) frameworks.

Given that our review includes a diverse range of study designs—including quantitative, qualitative, and mixed-methods studies—and aims to map the breadth of literature on psychosocial interventions in mental health and problems, we determined that the PCC framework was more appropriate. The PCC framework is recommended for scoping reviews by the Joanna Briggs Institute and allows for broader inclusion criteria, which aligns with our objective to identify gaps and trends in the existing research.

We have updated the Methods section to include the rationale for choosing PCC framework, as below (page 8, lines 130-133):

The inclusion and exclusion criteria were developed using the population-concept-context (PCC) framework, which is recommended for scoping reviews that aims to map key intervention concepts and identify gaps in the literature. The PCC framework is suitable for accommodating a wide range of study designs and does not require a comparison group, which aligns with our research objectives [27].

We hope this explanation clarifies our choice of framework and demonstrates its suitability for our study.

#### **Comment 3:**

*Present draft of search strategy to be used for at least one electronic database, including planned limits, such that it could be repeated.*

**Response:**

To facilitate transparency and reproducibility of the review process, the full search strategy for the Scopus database is already included in **Supplementary Appendix S1**. This includes search terms and keywords used, Boolean operators applied, filters and limits set, date range of the search

We have also updated the Methods section to make it explicit the location of the sample strategy and what it contains, as given below (page 10, lines 174-175):

The comprehensive search strategy to be used for Scopus database is provided in S1 Appendix, including all search terms, Boolean operators, and limits applied*.*

Please let us know if further details are required or if there were specific aspects you would like us to expand upon.

#### **Comment 4:**

*List and define all outcomes for which data will be sought, including prioritization of main and additional outcomes, with rationale.*

**Response:**

In our review, the effectiveness outcome data will be extracted for variables related to mental health and/or mental problems, and implementation outcome data will be extracted for domains such as acceptability, feasibility, fidelity and others as proposed by Proctor et al (2011). We have revised the Data extraction section of manuscript and S2 Appendix to include details of outcomes as follows:

Manuscript (page 12, lines 216-219):

…types of effectiveness outcomes assessed for mental health and/or mental problems (as defined in the concept section); types of implementation outcomes evaluated (as defined by Proctor et al. [22]); and key findings for each of these outcomes, including relevant participant verbatims where applicable (see S2 Appendix for details).

S2 Appendix (supporting information document, line 20)

Type of effectiveness outcome assessed for mental health (e.g., well-being, psychological distress, and/or determinants of mental health such as mindfulness, emotional regulation, critical thinking, self-efficacy), and/ or mental problems (such as depression, anxiety, burnout, and post-traumatic stress disorder), quantitative and/or qualitative outcome measure used, key findings on measure, relevant participant verbatims

Type of implementation outcome assessed (i.e. acceptability, feasibility, fidelity, cost, adoption, appropriateness, penetration), quantitative and/or qualitative outcome measure used, key findings on measure, relevant participant verbatims

#### **Comment 5:**

*How do the authors mean by this statement: "The extracted data will be synthesized using a mixed methods approach, combining narrative and thematic synthesis." Is synthesizing the extracted data using narrative and thematic approach make it a mixed method approach?*

**Response:**

Thank you for seeking clarification. In our scoping review, we define a mixed methods synthesis as the integration of both quantitative and qualitative data to provide a comprehensive analysis of the research topic.

- **Narrative Synthesis:** This approach involves summarizing and explaining the findings of multiple quantitative studies in a descriptive manner. It allows us to identify patterns, relationships, and trends across the quantitative data extracted, such as intervention effectiveness and measured outcomes.
- **Thematic Synthesis:** This method is used to analyze qualitative data from studies that include qualitative findings or components. Through thematic analysis, we can identify common themes, perceptions, and experiences related to the interventions.

By combining these two synthesis methods, we effectively integrate quantitative results with qualitative insights, which enriches our understanding of both the measurable effects and the contextual factors influencing the interventions.

We have revised the Data analysis section to clarify this, as below (page 12, lines 227-230):

The extracted data will be synthesized using mixed methods approach that integrates narrative synthesis of quantitative data with thematic synthesis of qualitative findings. This combined approach enables a comprehensive understanding of the interventions' effectiveness and factors affecting their implementation.

We trust that this explanation clarifies how the use of narrative and thematic synthesis constitutes a mixed methods approach in our review.
